# Junior doctors' experiences of the medical internship: a qualitative study

**DOI:** 10.5116/ijme.6229.d795

**Published:** 2022-03-23

**Authors:** Yvonne Carlsson, Anna Nilsdotter, Stefan Bergman, Matilda Liljedahl

**Affiliations:** 1 General Medicine, School of Public Health and Community Medicine, Institute of Medicine, Sahlgrenska Academy, University of Gothenburg, Gothenburg, Sweden; 2 Department of Orthopaedics, Sahlgrenska University Hospital, Gothenburg, Sweden; 3 Department of Oncology, Sahlgrenska University Hospital, Gothenburg, Sweden

**Keywords:** Introductory service, junior doctor, medical internship, postgraduate education, transition

## Abstract

**Objectives:**

This study aimed to explore medical interns' experiences of medical internships.

**Methods:**

Situated in an interpretivist paradigm, a qualitative study was carried out to explore medical interns' experiences of the internship. Invitations to participate were sent via email to medical interns currently in their last six months of internship. The first ones to respond were included. The study sample comprised twelve participants, of whom seven were women. Data were collected through individual, semi-structured and in-depth interviews with volunteering medical interns from three different hospital sites. Data were transcribed verbatim and analysed through qualitative content analysis, generating overarching themes.

**Results:**

Four main themes were identified in our data. The interns felt increasingly comfortable as doctors ('finding one's feet') by taking responsibility for patients while receiving necessary help and assistance ('a doctor with support'). Although appreciative of getting an overview of the healthcare organisation ('healthcare sightseeing'), interns were exhausted by repeatedly changing workplaces and felt stuck in a rigid framework ('stuck at the zoo').

**Conclusions:**

In contrast to previous studies, this study shows that the transition from medical school to clinical work as a professional does not necessarily have to be characterised by stress and mental exhaustion but can, with extensive support, provide a fruitful opportunity for medical interns to grow into their roles as doctors. However, there is still unutilised potential for the medical internship to act as a powerful catalyser for learning, which educators and programme directors need to consider.

## Introduction

In Sweden, graduation from the five-and-a-half-year medical programme is followed by a minimum of one-and-a-half years of postgraduate service: the medical internship (in Swedish, 'allmäntjänstgöring' or 'AT'). In many countries, including Sweden, a medical internship is a way of bridging the gap between medical school and specialty training.^1^ It is commonly defined as the period following undergraduate medical education and before becoming a fully licenced doctor, consisting of clinical work under supervision in accredited positions in both hospital and primary care settings and generally comprise one to two years of clinical duty.^2^ According to the National Board of Health and Welfare in Sweden, a medical internship should serve the purpose of introducing the newly graduated doctor to clinical work by allowing him or her to practice and develop knowledge and experiences based on what was gained during medical school.^3^ Albeit differently organised in different countries, many studies show that the transition from being a medical student to working as a medical doctor is intense and stressful.^2^^, ^^4^^-^^6^ International studies have shown high levels of burnout among junior doctors and report that they experience the transition as physically, mentally and emotionally exhausting.^7^ Additionally, newly qualified doctors have expressed not feeling prepared to start clinical work.^8^ Previous research has also established that junior doctors experience a lack of protected study time and formal educational curriculum^4^ and a tension between learning and working.^9^

The Swedish medical internship is regulated by the National Board of Health and Welfare, according to which the internship should provide prerequisites for the subsequent postgraduate residency (also known as specialist training). Although regulated nationally, the internship is managed at the hospital level. This means that the hospitals are responsible for the educational content during the internship as well as for assessing that each intern fulfils the national requirements to pass the internship to become a licenced doctor. The internship includes four to six months of internal medicine, four to six months of surgery and orthopaedics, three months of psychiatry and six months of primary healthcare. The rotation in primary healthcare must be the very last rotation of the internship, whereas the order of the other rotations can vary. Although having a dual focus on clinical work and formal education, most of the internship takes place in a clinical setting where interns work as medical doctors. Interns are included in the regular schedule of each workplace and are expected to contribute to the daily work of a doctor, including tasks such as admitting and discharging patients, performing physical examinations, prescribing medicine, writing referrals and patient notes, consulting specialists, and participating in ward rounds. Approximately half a day per week is dedicated to formal learning, including lectures, presentations, seminars and literature readings. Additionally, interns have a designated mentor with access to structured supervision and clinical feedback on a regular basis. Publicly funded hospitals are responsible for providing fully salaried positions for medical interns, and approximately 60 hospitals, i.e., the majority of all hospitals in Sweden,^10^ offer internship positions. Due to a lack of available positions, there is currently an average waiting time of 10.6 months from graduation until the start of the internship.^11^ During this time, many graduates work as junior doctors with clinical supervision but generally without further education, such as courses, structured supervision, lectures, etc.

A yearly national evaluation investigates interns' satisfaction with the medical internship in Sweden; according to this survey, the interns generally express high satisfaction.^12^ This contradicts previous literature from the international context where internships, from the interns' perspective, are usually described in pejorative terms. It would therefore be highly interesting to explore the Swedish medical internship as it, to date, has not been investigated in a qualitative manner, surpassing the more basic level of satisfaction. Consequently, this study aimed to explore medical interns' experiences of the medical internship.

## Methods

### Study design and participants

The study was conducted within an interpretivist paradigm, meaning that knowledge is understood as mutually constructed between the researcher and the participant.^13^ This approach is appropriate when exploring human beings' experiences, thoughts and perceptions with the goal of understanding and describing a phenomenon rather than trying to predict it.^14^ In line with the interpretivist tradition, the study took to on a qualitative approach. Accordingly, we did not expect to generate generalisable results but instead to describe individuals' own experiences in a deep way enabling questions of "how" and "why" to be answered.^15^^,^^16^ We employed a qualitative description design as we sought to describe medical interns' experiences in a language familiar to them.^17^ This design is particularly useful when the aim is to study individual experiences, since it allows the researcher to stay close to the data to produce a straight description of the phenomenon. Importantly, this does not equal an absence of interpretation, as no data speaks for itself.^18^ To analyse the data, we applied qualitative content analysis, the suggested analysis strategy in qualitative descriptive studies.^17^

Medical interns from two regional hospitals and one university hospital were invited as participants in the study. First, approval to invite medical interns to participate was received from the employer (head of medical interns). Then, invitations to participate were sent via email to medical interns currently in their last six months of internship, i.e., during their primary care rotation. The first ones to respond were included in the study, hence convenience sampling was employed.^19^ Further, the medical intern's willingness to share experiences was considered a useful starting point of the interview. Twelve medical interns were included, of whom seven were women. Ages ranged from 27 to 51 (median 29), and all but two had received their undergraduate training in Sweden. The gender, age and site of undergraduate training of our participants were on average coherent with medical interns in Sweden in general.^20^^, ^^21^

The study was conducted in accordance with the Declaration of Helsinki.^22^ Ethical approval was sought from the Regional Ethical Review Board in Gothenburg, who approved the study design, including sampling, data collection and data analysis as well as management of data and personal records. Oral and written information was given to all participants before the study. It was stressed that participation was voluntary and that the participants could, at any time, withdraw their participation. Written informed consent was obtained from all participants prior to the interviews.

### Data collection

Individual and semi-structured interviews were performed by ML, shaped by an interview guide based on the aim of the study and a literature search performed when initiating the research project.^23^ The interview guide is enclosed as Appendix. Opening questions (e.g., What do you do as a medical intern? How have you perceived the role you have been given in the clinical workplace?) were followed by probing questions (e.g., Can you explain what you mean by that?). Interviews were audio-recorded and, in most cases, held at the interviewee's workplace. Two interviews were held over the telephone due to geographical constraints. Interviews lasted between 34 and 49 minutes and were transcribed verbatim by YC.

### Data analysis

Data were subject to qualitative content analysis according to Elo and Kyngäs.^24^ An inductive stance was taken; that is, the analysis was data-driven rather than based on a pre-existing theory or framework.^25^ Interview transcripts were read through for familiarisation, followed by a systematic highlighting of words, sentences and paragraphs corresponding to the research question. Through this, a set of meaning units was created and in turn, condensed while preserving their essence. Condensed meaning units with related content were then collated into categories. Following this, categories were grouped under higher-order headings to create overarching themes. Some themes were merged, and some were discarded due to insufficient data supporting them. The goal was to reach internal homogeneity, i.e., data within one theme showing similarity and shared meaning, and external heterogeneity, i.e., separate themes being clearly distinct.^26^ Each theme and its categories were continuously verified in relation to the data, to assure a coherent pattern within each theme as well as to double-check that identified quotes were not decontextualised. ML and YC performed all steps of the analysis in parallel, verifying categories and themes regularly with all members of the research team. The purpose of these discussions was not to reach strict consensus, but to allow us to look at the data from multiple perspectives. The aim and the research question were used to guide data collection and analysis. Furthermore, participants of both genders and from different hospital sites were included to achieve a diverse sample.

## Results

This paper draws upon the experiences of twelve interviewees working as medical interns at the end of their internship. Four major themes were identified, each theme containing several categories. Themes were 'finding one's feet', 'a doctor with support', 'healthcare sightseeing' and 'stuck at the zoo'.  Themes and corresponding categories are outlined in Table 1. Quotations have been provided to illustrate the four themes, providing the reader with an opportunity to evaluate their appropriateness.

### Finding one's feet

The interns reported that they started settling into their roles during the internship. Acting as a doctor became less intimidating, and interns expressed feeling increasingly comfortable and confident taking on a day's work. The interns expressed that through daily clinical work, their knowledge deepened. It became a habit to diagnose and treat common conditions, and they could better differentiate a critically ill patient from a healthy one. This enhanced medical knowledge was perceived as increasing their trustworthiness in relation to patients and colleagues and making them feel both empowered and more independent as doctors. As one participant said, medical school had been taken to a new level:

'I know about many things after medical school. I know about common diagnoses, and maybe I've even palpated a peritonitis, if I've been lucky, and maybe inserted a nasogastric tube and things like that. But to make the decision to insert that tube and the decision to start operating … there is a certain level of difference there' (No. 9, Male)

**Table 1:** Themes and corresponding categories

Theme	Category
Finding one's feet	Settling into the role
	An increased medical knowledge
	Producing healthcare
	A new focus
	Becoming a decision maker
	A need for responsibility
A doctor with support	A safe structure
	Lower expectations and demands
	Allowed to ask
	The internship equals education
	The support can be limiting
Healthcare sightseeing	Grasping the organisation
	A varying role
	An anonymous guest worker
	Always new at work
Stuck at the zoo	It's agreeable
	It is what you make of it
	Having a low rank
	Life turns inflexible
	In charge of one's own development
	Ready to move on

In this new role as doctor, they experienced having to make decisions of their own, determining treatments and remedies, estimating risks, as well as making decisions about oneself and when to ask a superior for help or decide to manage on their own. Being trusted to take on the responsibility of a decision-maker was regarded as important in enabling them to learn and grow as doctors:

'It was not as if I was abandoned, but I was allowed to be in charge of the patients, and I assessed 19 patients that shift. [...] I had never felt that pushed and supported ever before, as when someone actually gives you the responsibility' (No. 11, Female)

Although they expressed that they learned from 'getting the job done' and that they understood that someone who becomes a doctor must know how to write referrals, admit and discharge patients and take care of administrative tasks, the interns struggled to find a balance between 'producing healthcare' and making sure they received appropriate education. They repeatedly reported tensions between working and learning:

'One writes discharge notes (epicrisis), and that is great for everyone else, but myself, I don't learn anything from it' (No. 1, Female)

### A doctor with support

According to the participants, being a medical intern came with various forms of support. On a structural level, the interns experienced having an overarching safety net provided by the organisation and the responsible stakeholders at each hospital. Supervisors and programme directors were close at hand if interns encountered problems:

'We know exactly whom to turn to with our concerns and there's a lot of support from the head of interns' (No. 11, Female)

At the workplace, the interns felt assured that it was both legitimate and expected of them to call for assistance and frequently ask questions. They expressed that it was acceptable to feel unsure and hesitant; they felt comfortable being novices. Moreover, they noted that the demands and expectations placed upon them were lower compared to their senior colleagues. For instance, they knew that they were allowed to have a slower work pace, conducting thorough medical histories and looking up doses and contraindications; this provided a 'win-win situation':

'Patients know that since you are an intern […] you make mistakes, but they are also very happy and satisfied when they meet an intern, because we have much more time and we want to do much more, and we are super meticulous' (No. 2, Male)

One participant thought that having a name badge with 'medical intern' written on it provided a sense of security, in terms of not receiving full responsibility for patients, and helped in not merely being seen as a caregiver, but as a learner in need of supervision and education. Also, the interns said this identification justified attending potential learning opportunities not necessarily related to their assigned patients:

'As an intern, you can be […] more frequently asked when something happens; "Do you want to insert the nasogastric tube?", "Do you want to attend this gastroscopy?", "Do you want to join in on this ultrasound?"' (No. 8, Female)

### Healthcare sightseeing

The interns described how the medical internship meant an ever-changing workplace. Overall, they appreciated the rotations on several wards, emergency departments and clinics, both in outpatient and inpatient settings. They expressed that this kind of 'sightseeing' gave them an opportunity to get an overview of and grasp the organisation of the healthcare system. However, rotating from one department to another had negative consequences as well, and the interns expressed feeling anonymous, like 'guest workers':

'At worst, I've felt that nobody knows who I am or why I'm there, and then you become a healthcare-producer more than anything else. You know that you're going to be there for two weeks, and it feels like no one cares about … me!' (No. 7, Female)

Additionally, the roles allocated to the interns differed. They could range from almost being alone in the emergency department during a night shift to watching surgeons performing appendectomies and 'hardly being allowed to breathe'. Put differently, the interns were sometimes assigned substantial responsibility and at other times none. Another aspect was the exhaustion and tiredness resulting from constantly changing departments. Much time and energy were spent on understanding local routines and customs when starting a new rotation:

'The actual problem, except that you have to be happy and friendly every Monday, is that there is a lot of logistics. "Now I've finished this paper, should I put it in this folder or in that folder?" [...], and it hinders the theoretical learning. It's all about learning routines' (No. 10, Male)

### Stuck at the zoo

The interns experienced the internship as a 'win-win', learning and gaining experience while working and receiving a salary. Nonetheless, they acknowledged the internship as mandatory and knew that they had to complete all predetermined clinical rotations to become a licenced doctor, thus, the 'stuck at the zoo' analogy. They felt stuck in a predetermined framework, yet it was at the same time comfortable and satisfactory:

'There are actually very few professions where you've got that luxury, to be allowed to work while receiving supervision' (No. 5, Male)

The interns noted they did not have the same flexibility as other employees to take time off or make changes in their schedules. Although inflexible, the internship was likewise seen as a time when one could make use of the framework and profit from the circumstances. They expressed that there was space to learn and do more during the internship to make the most of it, but there was also an option to choose the path of least resistance:

'One gets to choose whether to take on a lot and aim to learn, or to somehow just survive the internship. […] One can take very little responsibility, or one can take a lot' (No. 4, Female)

Further, the interns felt that they were low-ranking, with patients sometimes regarding them as beginners and superior doctors occasionally thinking they could use the interns for the 'dirty work'. Nevertheless, the interns emphasised the need to take responsibility for one's own learning. To avoid being used by the system or becoming a victim of circumstances, they felt that they had to stay vigilant and aware of their rights and obligations. Once they approached the 'finish line' and looked back at their time as interns, they felt that it had been a good experience, that they had learned a significant amount, but likewise ready for the next step:

'I feel quite done with being an intern. I wouldn't like to keep doing this that much longer, because I do not think it would give me more in this form, more than knowledge of course, but … I want to move on' (No. 7, Female)

## Discussion

This qualitative study reports on how medical interns experiences the medical internship. The four themes provide a narrative of the internship, highlighting the benefits and the opportunities it allows, but also the drawbacks; taken together, these aspects had considerable impact on the medical interns and their transition to clinical work.

### The internship as a transition

A major theme in the findings is how the internship enabled the interns to feel comfortable and safe in their new role as doctors—it allowed them to 'find their feet'. Overall, they seemed grateful for the support they received, and the lowered demands placed upon them. The internship provided an opportunity to 'try out' working as a doctor and put their knowledge into practice. This is in line with the recommendations of the National Board of Health and Welfare in Sweden, emphasising that the internship should bridge the gap and allow interns to apply previously learned knowledge. International studies have shown that similar qualities, notably aspects of real-time on-the-job support^27^ and experiential practice,^28^ are components of a successful transition to clinical work.

Although occasionally experienced as tiring and exhausting, the internship was, interestingly, not depicted as a major source of anxiety or fear. Reports of heavy stress and mental exhaustion were absent in our data. This finding contradicts the reports from comparable international studies, where the transition is often referred to as stressful and mentally overwhelming.^5^^,^^7^ A possible explanation for this may be the clearly defined support and the lowered demands placed upon the interns. They expressed that they were always allowed, often expected, to ask questions, seek assistance and confirm plans with their tutors. This resonates with international studies that have described a lack of support related to patient assessment as a crucial factor to mental distress among junior doctors.^6^ Similarly, previous studies have demonstrated that a positive learning climate protects against mental exhaustion.^29^

An additional explanation could be that many medical interns in Sweden have worked on temporary contracts before being admitted as medical interns. This prior exposure to clinical work might alleviate possible anxious or fearful experiences linked to the medical internship. Nonetheless, the interns repeatedly expressed how the internship specifically provided opportunities to take responsibility for patients, while simultaneously being a safe learning environment for them as junior doctors. In that sense, we argue that the medical internship, at least to some extent, found what O'Brien called the 'sweet spot between challenge and support’.^30^

### Learning versus working

Although acknowledging that one must work to learn, the interns tended to look at learning and working as two separate concepts. They found that administrative tasks often took time away from learning, and while happily engaging in patient-related work, they still expected it to come with some sort of educational purpose. This tension between learning and working resonates with the previous work of Skipper et al.^31^ In their study, residents saw themselves as service providers and found many tasks to be without educational value. Additionally, the term 'service' has been shown to have a negative connotation and is frequently used to describe experiences interfering with learning^32^ which was also the case in our study.

In the literature, nonetheless, it is well established that postgraduate training is built upon learning from work activities.^9^^,^^33^ Activities, such as doing rounds, taking a patient's medical history and assessing and discharging patients, have thus been argued as making up the entire foundation of workplace learning. Seemingly, interns' perception of learning differs from how learning is known to take place. This tendency to perceive work, or service, as taking time from learning, is disadvantageous in a healthcare setting where junior doctors need to learn from experiences gained at work. Therefore, the perception of how learning happens needs to be challenged, and work activities need to be acknowledged as learning opportunities in their own right. Yet, for work activities to act as learning opportunities, they need to be designed with the individual learner's needs in mind.^34^^,^^35^ Neglecting to consider what kind of activities medical interns should engage in and instead randomly placing a learner in a hospital-based environment and expecting him or her to efficiently work there is counterproductive. Thus, we argue that it is of value to broaden the perception of how learning happens, in combination with assessing training doctors' individual needs for learning, to relieve this unfavourable tension.

Even though medical interns reported having extensive support from supervisors and other medical staff in terms of assessing, diagnosing and treating patients, they did not report any substantial support in their own learning. It would therefore be naïve to expect medical interns to resolve the learning-versus-work tension on their own. Rather, interns need assistance from supervisors and mentors in balancing learning activities. We argue that there is unutilised potential for the medical internship to act as a powerful catalyser for learning, which educators and programme directors need to consider.

### Limitations

Our study was conducted in a single context and health economy. Thus, a transfer of the findings beyond this specific environment must be made carefully. Although the sample was small, data was considered saturated based on the appreciation that very limited new information was being uncovered during the last two interviews. This study was based on interviews, meaning a loss of information during the audio recording and data transcription, such as the participant's body language, gestures, facial mimic, intonations and hesitations.^36^ We included interns from both regional and university hospitals; however, no significant difference between the locations was noticed in the data. The diversity of the research team, with both junior (ML, YC) and senior (AN, SB) doctors, of whom one is a former head of interns (AN), enabled us to challenge the interpretations of the data as we brought in different perspectives. It was arguably a limitation that the research team exclusively consisted of medical doctors. However, an outsider perspective was achieved through the main analyst (YC) not having any previous experience of the medical internship when analysing the data. The team included researchers with expertise in qualitative research (SB, ML), enabling high-quality analysis to be done. Although the participants are owners of their experiences, we as investigators see ourselves as co-constructors of knowledge, in accordance with the interpretivist approach.^37^ Consequently, the interviews and the analysis have been guided by our curiosity, beliefs and preconceptions.

## Conclusions

This study explored medical interns' experiences of the internship through qualitative interviews analysed with content analysis. In summary, this study has shown that the internship constituted an opportunity for the interns to 'find their feet' and to grow as doctors. The findings argue for providing medical interns with extensive support, possibly reducing levels of stress and negative experiences of the internship. Overall, these findings offer interpretations of why the medical internship studied here can be considered a successful means to introduce newly graduated doctors to clinical work. Nonetheless, our results imply that it is fundamental that work-based activities are not merely seen as work or service, but as essential learning opportunities and accordingly designed as such. To further understand the needs of medical interns, future research could attempt to go beyond the mere experiences of the medical internship and explore how medical interns and other stakeholders conceptualise the meaning of a medical internship.

### Acknowledgements

The authors would like to thank all participants for willingly sharing their thoughts, reflections and experiences. The study was supported by grants provided by the Gothenburg Society of Medicine, as well as by the Swedish state under the agreement between the Swedish government, the country councils (the ALF agreement).

### Conflict of Interest

The authors declare that they have no conflict of interest.

## Supplementary material

Supplementary file 1 Appendix. Interview guide
