# Supplementary material for: Junior doctors' experiences of the medical internship: a qualitative study
Source: Int J Med Educ. 2022 Mar 23;13:66–73. doi: 10.5116/ijme.6229.d795 (PMC9017508; doi:10.5116/ijme.6229.d795)
Supplement: Supplementary file 1 — Appendix. Interview guide [file ijme-13-66-S1.pdf]

Appendix

Interview guide

- According to you, what is a medical intern?
- According to you, what does it mean to be a medical intern?
- What do you do as a medical intern?
- How do you take on a day's work?
- What is the role of an intern?
- How does the role of a medical intern differ from other roles, for example from being a junior doctor before the internship, or a junior doctor after the internship?
- According to you, what opportunities are there in the role of being an intern?
- According to you, what limitations are there in the role of being an intern?
- If any, what role does the medical internship play in relation to your professional development?
- What meaning does the medical internship have for you?
- In your opinion, what function does the internship fill – now, in the future, personally?
- Have your thoughts on the internship changed during the internship?
- What are your thoughts on the meaning of the internship in relation to your future career as a doc-tor?
- Is there anything more you would like to add?
